# Editorial: Prompts: the double-edged sword using AI

**DOI:** 10.3389/frai.2025.1756343

**Published:** 2025-12-16

**Authors:** Jordi Vallverdú, Rafal Rzepka, Alger Sans Pinillos

**Affiliations:** 1 Philosophy Department, Autonomous University of Barcelona, Barcelona, Spain; 2 Language Media Lab, Faculty of Information Science and Technology, Sapporo, Japan; 3 Department of Computer Applications in Science and Engineering (CASE) Barcelona Supercomputing Center, Barcelona, Spain

**Keywords:** creativity, dual-use, epistemology, ethics, multimodality, optimization, prompting

## 1 Context and motivation

The impetus for this Research Topic emerged from our collective work as editors in AI ethics, computational creativity, cognitive science, and human-AI interaction. In recent years, we have observed that prompting is no longer a marginal technical detail but a central component of AI reasoning and user experience. Research into prompting has expanded to include causal modeling, epistemology, creativity, and safety. This evolution is tightly connected to the rise of large-scale foundation models, which concentrate capabilities and risks in general-purpose architectures that are adapted to a wide range of downstream tasks (e.g., Bommasani et al., 2021).

Several conceptual developments have helped shape the intellectual background of this Research Topic. For example, analyses of how prompts structure causal narratives in AI systems, as explored in Vallverdú's (2025) *Prompting Causal Events*, contributed to the early recognition that prompts act as cognitive scaffolds, organizing how models simulate explanations and relate events. Similarly, discussions of meaning-making in disembodied generative systems—such as Vallverdú and Redondo (2025) study on how LLMs construct understanding without embodiment—highlighted fundamental challenges in aligning user intentions with systems that lack lived experience. These works did not dictate the scope of the issue but informed the broader conceptual landscape that motivated us to curate a collection addressing prompting from multiple disciplinary angles.

Beyond these conceptual motivations, our collective expertise as Topic Editors also shaped the design of this Research Topic. Drawing from Rzepka's long-standing work in affective computing, computational linguistics, and human–machine interaction (e.g., Higuchi et al., 2008; Ptaszynski et al., 2009), and Sans Pinillos' research on abductive reasoning and the ethics of AI systems (e.g., Sans and Casacuberta, 2018), along with its implications for dual-use technologies (e.g., Sans Pinillos and Vallverdú, 2025), we aimed to push the conversation one step further. Our intention was to move beyond the technical mechanics of prompting and to explore its broader epistemic, social, and creative implications. This interdisciplinary perspective allowed us to curate contributions that not only analyze prompting as it exists today but also envision how it may evolve in the near future, encouraging the innovative and responsible use of generative technologies.

At a more global level, prompting itself is emerging as a new layer of computational technology. Recent work in natural language processing has conceptualized prompting as a new programming paradigm for large models, in which natural language becomes a high-level control language for pre-trained systems (e.g., Liu et al., 2022; White et al., 2023). From this perspective, prompts function less as ad hoc queries and more as an interface technology comparable to an operating system or an API. Treating prompting as such a foundational layer motivates the need for careful analysis of its epistemic, ethical, and creative dimensions, which is precisely the aim of the present Research Topic.

## 2 Prompting as technical optimization

The contribution *GAAPO: genetic algorithmic applied to prompt optimization* by Sécheresse et al. illustrates a growing methodological trend: using computational tools to systematically optimize prompts. Their genetic algorithm demonstrates that prompt engineering can be automated, revealing formulations that significantly enhance performance. This raises important questions. As prompts become optimized by machines rather than humans, do we risk separating operational effectiveness from human interpretability? While automated discovery expands the expressive power of LLMs, it may also widen the gap between user understanding and model behavior. This tension is emblematic of the technical duality of prompting: it is both an accessible interface and a sophisticated control surface.

## 3 Prompting and ethical responsibility

Farnós et al. in *Ethical prompting: toward strategies for rapid and inclusive assistance in dual-use AI systems*, analyze prompting through the lens of ethics and governance. Prompts can enhance safety by enabling explicit constraint formulations, but they can also inadvertently bypass safeguards when poorly specified or intentionally manipulated. As models proliferate in sensitive or high-impact contexts, prompting becomes an ethical act, not merely an operational one. The authors compellingly argue for developing strategies that enable rapid and useful assistance while maintaining inclusivity and avoiding misuse. This aligns with broader discussions in AI governance: prompting increasingly resembles a form of literacy, where understandings of risk, bias, and responsibility must be integrated with technical competence, and where large language models are increasingly analyzed as sociotechnical systems whose scale and opacity raise concerns about bias, misuse, and environmental impact (e.g., Bender et al., 2021; Bommasani et al., 2021).

## 4 Prompting and creative constraints

Casacuberta and Guersenzvaig in their article *Disembodied creativity in generative AI: prima facie challenges and limitations of prompting in creative practice*, examine prompting within artistic contexts. Generative systems enable new forms of creative production; however, the language-based nature of prompting introduces constraints. Much of creative practice relies on tacit, embodied, or material knowledge—elements that are difficult or impossible to encode as text. Their analysis shows that prompting can simultaneously open and limit creative spaces. Although generative models provide new expressive tools, they also risk standardizing artistic output around what is easily described. This reflects the deeper challenge of disembodied generative systems: they simulate meaning and creativity through linguistic coherence rather than experiential grounding.

## 5 Toward a unified understanding of prompting

Across the contributions, three unifying themes emerge:

1. Prompts as operational controls Prompts determine how systems behave, which capabilities are activated, and how models respond to uncertainty.
2. Prompts as epistemic structures Prompts shape what the model considers relevant, how it assembles explanations, and how it constructs apparent meaning. These dynamics resonate with earlier reflections on causal prompting and disembodied understanding.
3. Prompts as socio-ethical instruments Because prompting can amplify or reduce risks, its role in dual-use scenarios must be carefully managed. Ethical prompting becomes indispensable in domains where trust, safety, and fairness matter.

These themes clarify why prompting is inherently “double-edged.” It democratizes AI access while introducing new vulnerabilities. It enhances creativity while imposing linguistic constraints. It provides powerful control over generative systems while making accountability more complex.

## 6 Future directions

Looking ahead, several research avenues appear especially urgent as prompting becomes further embedded in research, education, creativity, governance, and everyday technological practices. One key priority is the development of prompt literacy, ensuring that users not only learn how to obtain effective outputs but also understand the ethical, cultural, and epistemic dimensions embedded in each interaction with generative systems. Closely related to this is the need for explainable prompting: advancing methods that reveal why certain prompts succeed or fail, how optimized prompts differ from human-generated ones, and how users can maintain agency when interacting with increasingly opaque systems.

Another important direction concerns multimodal and embodied prompting. Future AI systems may integrate textual instructions with perceptual, sensorimotor, or environmental cues, thereby reducing the overreliance on language alone and enabling richer forms of interaction. At the same time, increasing attention must be given to cultural and linguistic pluralism, as prompting practices vary significantly across languages and communities. Understanding these differences is vital not only for fairness and accessibility but also for preserving epistemic diversity in AI-mediated reasoning.

Finally, prompting is poised to play a growing role in governmental and institutional decision-making. As public administrations explore the integration of AI-assisted tools into their workflows, prompting could support faster and more informed decisions, provided that transparency, accountability, and robust ethical safeguards are maintained. Together, these directions highlight that prompting is no longer a minor interface technique but a central component of the future human–AI ecosystem, one whose development requires careful interdisciplinary attention.

## 7 Conclusion

This Research Topic offers an integrated view of prompting as a critical practice in modern artificial intelligence. By examining technical optimization, ethical responsibility, and creative expression, the contributions highlight both the promise and perils of natural language prompting. We thank all the authors and reviewers for their contributions to this interdisciplinary dialogue. We hope this Research Topic inspires further research that advances the responsible, creative, and thoughtful use of prompting in AI systems.
